# Applying the Ecology Model to Perinatal Medicine: From a Regional Population-Based Study

**DOI:** 10.1155/2011/587390

**Published:** 2011-07-25

**Authors:** Syuichi Tokunaga, Hiroshi Sameshima, Tsuyomu Ikenoue

**Affiliations:** Department of Obstetrics and Gynecology, Faculty of Medicine, University of Miyazaki, 5200 Kihara, Kiyotake, Miyazaki 889-1692, Japan

## Abstract

*Objective*. Ecology model is useful to provide a framework for organizing medical care. We aimed to see if the ecology model is applicable to perinatal care in Japan. *Methods*. On a population-based approach, we had 53,461 deliveries in Miyazaki from 2001 to 2005. In comparison, we used all of the 106,613 deliveries in Tokyo in 2009. Women were divided into 4 grades by risk-allocation criteria and their proportion was expressed per 1,000 women to apply to the model and to delineate the ecology curve. The perinatal mortality was compared by Chi-square test. *Results*. We found remarkable similarity in ecology curves between the original ecology models and that representing Miyazaki perinatal data. However, the curve representing Tokyo was different from the original one. Besides, the perinatal mortality was significantly lower in Miyazaki (4.40/1,000) than in Tokyo (5.06/1,000). *Conclusion*. Applying the ecology model to perinatal care is useful with improvement of perinatal outcome and it would provide an appropriate framework for organizing perinatal care.

## 1. Introduction

The ecology model of medical care was first introduced in 1961 [1]. In this original model concerning 1,000 adults at risk, 750 of these individuals feel sick, 250 consult a physician, 15 require specialized care, and 1 needs to be referred to a university medical center. A recent study to apply this model to modern medical care has shown some variation from the original one, but overall relationships are still stable for 40 years [2], suggesting that the original ecology model can be used as a standard framework for organizing health and medical care, medical education, and researches. 

Ecology is applicable to clinical medicine in which structure and dynamics among the medical personnel, disease prevalence, and health care system should be taken into consideration. For example, it will establish a balance between medical system providing all sites of care in a region and the portion of population who needs medical care according to the disease severity. Likewise, it also balances health care seeking behavior of people with the actual health service utilization. 

From the obstetric viewpoint, routine care for low-risk women belongs to primary care, some women with high-risk factors need specialized care requiring high-level perinatal centers, and some women need emergency transfer to tertiary centers due to severe complications of the mother and infant. However, application of the ecology model to obstetric care has not been performed using a population-based approach. Thus, we hypothesized that this ecology model is applicable to a system providing perinatal care in Japan, where primary caregivers deal with low-risk pregnancies and high-level centers take charge of high-risk pregnancies to improve good maternal and childhood outcomes. Japan is unique to have ethnically almost homogeneous population and to have a universal health care system that allows everyone to access freely to hospital. Besides, we have a standardized perinatal care system, in which each medical district of 1 million population has 1 tertiary center with several affiliated secondary centers, in which high-risk women are transferred from regional primary hospitals. If the ecology model is applicable to perinatal care, it will enable us to arrange an appropriate framework under the balance between the health care demands and supplying medical personnel.

## 2. Materials and Methods

This study was approved by the Institutional Review Board, Faculty of Medicine, University of Miyazaki. 

In 1997, we started a regional population-based study on all perinatal deaths in Miyazaki prefecture, where we have 10,000 deliveries per year in a population of 1 million. Details of the study have been reported elsewhere [3]. Briefly, we have 34 primary obstetric hospitals in which a total of 52 obstetricians work regularly, 7 secondary perinatal centers with 29 obstetricians, and 1 tertiary center with 23 obstetricians. All but 2 primary hospitals have 30-minute access to nearby secondary centers. Low-risk women deliver mainly in the 34 primary hospitals. The 8 high-level (secondary and tertiary) centers deal mainly with high-risk pregnancies. We hold a peer-review audit conference twice a year to determine the causes and clinically associated factors of perinatal deaths [3]. Perinatal deaths consist of fetal death (at least 22 weeks gestation) and neonatal death (up to 4 weeks of life). Maternal death is the sum of direct and indirect obstetric deaths during pregnancy and until 6 weeks postpartum.

When antepartum high-risk factors are diagnosed, women are advised to visit high-level perinatal centers where they finally deliver their babies. High-risk factors include prenatal medical complications such as diabetes, obstetric complications such as hypertensive disorders, and fetal complications such as growth restriction. Additionally, some emergencies may occur and they are transferred to the 8 high-level centers. To determine the reasons for emergency transfer, a questionnaire was sent and, if necessary, we directly reviewed the medical charts and interviewed the physicians in charge. 

We used the above-mentioned data from 2001 to 2005 and applied them to the ecology model [1, 2]. For comparison, we used the perinatal data from Tokyo where only 30% of women deliver their babies in primary hospitals [4–6], the number of 30% is the lowest of Japan in 2009. The original model uses the number of persons per 1,000 citizens per month [1, 2]. Instead, we used all of the pregnant women and calculated the number per 1,000 pregnancies.

Prevalence of risk-allocated persons per 1,000 was compared by Chi-square test and *P* < 0.05 was considered statistically significant.

## 3. Results and Discussion

From 2001 to 2005, we had 53,461 deliveries and 235 perinatal deaths (4.40/1,000) including 158 fetal deaths and 77 neonatal deaths. The 34 primary hospitals dealt with 42,080 (787/1,000) low-risk deliveries, 7 secondary centers had 9,887 (185/1,000) deliveries with some high-risk factors, and 1 tertiary center had 1,494 (28/1,000) deliveries with the most high-risk factors. We also had 1,504 emergency transfers to high-level institutions (28/1,000), among which 192 (13%) were maternal indications and the remaining 1,312 (87%) were fetal indications. Among these cases, 89 required a university-setting care, which accounted for 1.7/1,000 of all deliveries (Table 1). The 7 secondary centers dealt with perinatal deaths most frequently (58% of fetal deaths and 51% of neonatal deaths) followed by 1 tertiary center (21% of fetal deaths and 44% of neonatal deaths) and 34 primary hospitals (21% of fetal deaths and 5% of neonatal deaths).

In Tokyo, they had 106,613 deliveries in 2009 [4–6]. Primary hospitals dealt with 30.1%, secondary centers had 49.5%, and 20 tertiary centers had 20.4% of deliveries. They had 1,558 (1.46/1,000) women required emergency maternal transfers to the university-settings to take care of their high-risk factors. Unfortunately, incidences of medical risk factors such as diabetes and hypertension during pregnancy are not reported in a regional population-based study from Tokyo, but we speculate that no significant differences exist in any regions in Japan because of our almost homogenous ethnic population and socioeconomical conditions. 

The average perinatal mortality rate of Tokyo was 5.06/1,000 during 2001 to 2005 [4–6], which was significantly higher than that of Miyazaki (4.40/1,000) (Table 1). The causes of perinatal deaths have not been revealed by a population-based approach from Tokyo, that prevented us from investigating the differences in causes of perinatal mortality and the differences in medical care levels in which unexplained perinatal deaths occurred.

Table 1 compared the prevalence of risk-allocated persons per 1,000. There was no statistical difference between the original data and the updated data 40 years later in the primary medical care (Chi-square test). The data representing perinatal care in Miyazaki were significantly different from the original one (*P* = 0.02), but when the study period was taken into consideration, the prevalence of Miyazaki data was no longer different from the revisited one in 2001. On the other hand, the data representing Tokyo were significantly different from those of the remaining 3 groups (*P* < 0.001).

The emergency maternal transfer rate to the university-settings was similar between Miyazaki (1.66/1,000) and Tokyo (1.46/1,000). However, overall maternal transfer rate to high-level centers was not available in Tokyo. The difference in the overall maternal transfers may contribute to some extent to the differences in perinatal mortality rates, which need further studies. 

**Figure 1:**
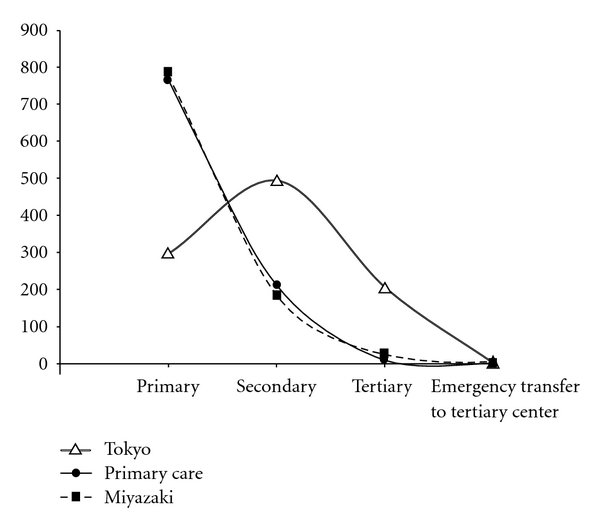
Ecology curves showing the relationship between patient number per 1,000 as a function of perinatal care levels. There is similarity between the combined original ecology curve (solid) and that for Miyazaki data (dotted). However, the curve derived from the Tokyo data (doublet) is apparently different from the original curve.

Since the prevalence of risk-allocated persons was not different between the original and updated data (Table 1), we used combined data to obtain the regression curve: The highest *r* ^2^ was obtained by the following equation: *Y* = 1845 − 1400∗*X* + 350∗*X* ^2^ − 29∗*X* ^3^, (*r* ^2^ = 0.998), where *Y* is the number of patients per 1,000 and *X* is the level of perinatal care such as primary = 1, secondary = 2, tertiary = 3, and emergency transfer to tertiary centers = 4.  Figure 1 illustrates the ecology curves showing the patient counts per 1,000 persons on the vertical axis, and 4 levels of perinatal care on the horizontal axis. The combined original ecology model showed a concave curve. The curve representing Miyazaki data was superimposable on the original curve, while the curve derived from the Tokyo data was apparently different from the previous 2 curves (Figure 1). 

The differences in ecology curves between Miyazaki and Tokyo are unexplained in the present study. One possibility is that the number of primary hospitals has been decreasing in Tokyo, while it is relatively stable in Miyazaki. Another possibility is that pregnant women's behavior is different in that women in Tokyo prefer high-level institutions to primary hospitals even though they do not have high-risk factors. Women in Miyazaki more likely follow the policy that low-risk women should be cared for in primary hospitals.

The specialized application of the ecology model to pregnant women has not been previously performed using a population-based approach, which would provide a useful framework for organizing health care, medical education, and research. We introduced a new concept of ecology curve (Figure 1) that shows the relationship between patient number per 1,000 as a function of perinatal care levels. Remarkably, the ecology curve representing Miyazaki perinatal care has changed little from the original curve representing primary care. This similarity may suggest that the system providing obstetric care could resemble that for primary care if high-risk women account for 20–25%. In the present study, the prevalence of high-risk pregnancies is estimated by the proportion of deliveries in high-level centers (21.3%), which includes both antepartum and intrapartum high-risk factors. It is compatible with a previous report showing that 15.3% of 4 million live births have obstetric and medical risk factors detected during the antepartum care [7]. Besides, 5 to 10% of low-risk pregnancies have some intrapartum risk factors [8]. 

Good childhood outcomes are achieved along with our perinatal risk-allocation system, where low-risk pregnancies are cared for by primary hospitals (80% of all deliveries) and high-risk pregnancies by high-level centers. Thus, we speculate that applying the original ecology model to the perinatal medicine is beneficial to provide a useful framework for organizing perinatal care system. If low-risk women bypass the primary hospitals to secondary or tertiary centers, the dynamics of this ecology model may be destroyed and perinatal outcome would become poorer. 

## 4. Conclusions

Our population-based data showed remarkable similarity between the prevalence of high-risk pregnancies and the prevalence of required medical care in the original ecology model, resulting in remarkable similarity in ecology curves as shown in Figure 1. Although the health care system for pregnant women is different from that of primary care medicine, this similarity may support the notion that the ecology model is applicable to a risk-allocated system for obstetric care to provide good perinatal outcome. These ecological standpoints are needed to organize an effective system for perinatal medicine in which health care demands and supplying medical personnel are balanced.

## Figures and Tables

**Table 1:** Prevalence of risk-allocated persons per 1,000 in each study.

	Original	Revisited	Miyazaki	Tokyo
Primary hoptital	750	783	787	301
Secondary center	235	195	185	494
Tertiary center	14	21	26	204
Emergency transfer to tertiary center	1	1	2	1
Total	1,000	1,000	1,000	1,000

Miyazaki versus Original, (*P* < 0.001, Chi square test).

Tokyo versus Original, Revisited, and Miyazaki, (*P* < 0.001, Chi square test)
